# Laboratory-Confirmed Respiratory Infections as Predictors of Hospital Admission for Myocardial Infarction and Stroke: Time-Series Analysis of English Data for 2004–2015

**DOI:** 10.1093/cid/cix1144

**Published:** 2018-01-06

**Authors:** Ruth Blackburn, Honxin Zhao, Richard Pebody, Andrew Hayward, Charlotte Warren-Gash

**Affiliations:** 1 Farr Institute of Health Informatics Research, University College London; 2 National Infection Service Colindale, Public Health England; 3 Faculty of Epidemiology and Population Health, London School of Hygiene and Tropical Medicine, United Kingdom

**Keywords:** myocardial infarction, stroke, respiratory infection, epidemiology

## Abstract

**Background:**

Acute respiratory infections are associated with increased risk of myocardial infarction (MI) and stroke; however, the role of different organisms is poorly characterized.

**Methods:**

Time-series analysis of English hospital admissions for MI and stroke (age-stratified: 45–64, 65–74, ≥75 years), laboratory-confirmed viral respiratory infections, and environmental data for 2004–2015. Weekly counts of admissions were modeled using multivariable Poisson regression with weekly counts of respiratory viruses (influenza, parainfluenza, rhinovirus, respiratory syncytial virus [RSV], adenovirus, or human metapneumovirus [HMPV]) investigated as predictors. We controlled for seasonality, long-term trends, and environmental factors.

**Results:**

Weekly hospital admissions in adults aged ≥45 years averaged 1347 (interquartile range [IQR], 1217–1541) for MI and 1175 (IQR, 1023–1395) for stroke. Respiratory infections ranged from 11 cases per week (IQR, 5–53) for influenza to 55 (IQR, 7–127) for rhinovirus. In the adjusted models, all viruses except parainfluenza were significantly associated with MI and ischemic stroke admissions in those aged ≥75. Among 65- to 74-year-olds, adenovirus, rhinovirus, and RSV were associated with MI but not ischemic stroke admissions. Respiratory infections were not associated with MI or ischemic stroke in people aged 45–64 years, nor with hemorrhagic stroke in any age group. An estimated 0.4%–5.7% of MI and ischemic stroke admissions may be attributable to respiratory infection.

**Conclusions:**

We identified small but strongly significant associations in the timing of respiratory infection (with HMPV, RSV, influenza, rhinovirus, and adenovirus) and MI or ischemic stroke hospitalizations in the elderly.

**Clinical Trials Registration:**

NCT02984280.

## Teaser

We investigated population-level associations between the timing of myocardial infarction (MI) or stroke hospital admissions and laboratory-confirmed respiratory infections. Infection with human metapneumovirus, respiratory syncytial virus, influenza, rhinovirus, and adenovirus was associated with increased ischemic stroke and MI risk in the elderly.

Globally, ischemic heart disease is the leading cause of death [1]. Over the past 2 decades, population growth and aging have facilitated a rising burden of ischemic heart disease despite falling levels of age-standardized incidence and case fatality of acute myocardial infarction (MI) in most world regions [1, 2]. Previous studies have shown a transient risk of acute vascular events including MI and cardiovascular deaths after clinically diagnosed acute respiratory infections (ARIs) from general practitioner or hospital records [3–5]. Influenza vaccine reduces the risk of major adverse cardiac events among people with existing cardiovascular disease [6]. However, few ARIs are laboratory-confirmed in health records, so assessing the burden of specific infections is often done indirectly using time-series models using laboratory surveillance datasets [7].

Time-series models show that influenza epidemics are associated with cardiovascular mortality in temperate, subtropical, and tropical climates [8–10]. UK studies have attributed a substantial burden of hospitalizations and deaths in older adults to both influenza and respiratory syncytial virus (RSV) [11, 12]. Seasonal all-cause mortality in the elderly has been attributed to multiple viruses including influenza, parainfluenza, RSV, and norovirus. Estimates from a Dutch study suggest that these viruses account for 6.8% of deaths in people aged ≥85 years, 4.4% in those aged 75–84 years, and 1.4% in people aged 65–74 years [13]. However, few studies examine the effects of a comprehensive range of respiratory viruses on specific cardiovascular end-points. Such studies are needed to inform vaccination and antiviral and antithrombotic strategies for high-risk patients and for health service planning, especially in settings with limited healthcare infrastructure [14].

In this ecological study we aimed to describe the temporal associations between different laboratory-confirmed respiratory viruses and hospital admissions for MI and stroke using laboratory-confirmed respiratory infections.

## METHODS

We undertook a time-series analysis of English national aggregated data on hospital admissions for MI and stroke (stratified by age: 45–64, 65–74, and ≥75 years), laboratory-confirmed viral respiratory infections, and environmental data for the period 1 April 2004 to 31 March 2015.

### Cardiovascular Outcomes

Counts of MI and stroke-associated admissions to English hospitals aggregated by week of admission (compatible with influenza surveillance weeks) and age were obtained from Hospital Episodes Statistics (HES) via National Health Service Digital.

MI and stroke events occurring in individuals aged ≥45 years were identified in HES data through records of the diagnosis at hospital discharge and classified according to the *International Classification of Diseases, Tenth Revision* codes for acute MI (I21 and I23) or for stroke comprising ischemic stroke (I63), hemorrhagic stroke reflecting intracerebral hemorrhage (I61), or subarachnoid hemorrhage (I60). We examined the impact of stratifying analyses for stroke admissions into ischemic vs hemorrhagic types. The date of admission was taken as the date of the vascular event.

### Respiratory Infections

Counts of respiratory viruses (influenza, parainfluenza, rhinovirus, RSV, adenovirus, or human metapneumovirus [HMPV]) aggregated by week of sample collection for people of all ages were obtained from LabBase [15], which captures all positive test results (ie, confirmed infections, hence no denominator data are available) reported by hospital laboratories in England. We included all respiratory samples, thus excluding test results (eg, gastrointestinal samples relating to adenovirus) that are not indicative of ARI. Samples submitted to LabBase come from both primary and secondary healthcare settings. HMPV appeared to be incompletely reported for early time points; thus, data for this virus were examined only for 2010 onward.

### Environmental Data

British Atmospheric Data Centre data on daily temperatures (minimum, mean, and maximum) in central England (approximately bordered by Bristol, Lancashire, and London) were obtained and aggregated by week. The MIDAS Land Surface Observation Stations dataset was used to obtain daily data on relative humidity across England, from which absolute humidity was estimated and aggregated by week [16].

### Statistical Analysis

Weekly counts of MI or stroke admissions (the primary outcomes) were modeled using Poisson regression with a scale parameter set to the Pearson χ^2^ statistic divided by the residual degrees of freedom to model overdispersion. Weekly counts of each virus were separately investigated as predictors in models incorporating long-term and seasonal variation as well as environmental data as covariates. We tested 4 approaches to modeling seasonal variation in MI and stroke: (1) categorical calendar quarter (ie, 4 quarters × 11 years); (2) month; (3) Fourier terms (plus a linear term for year); or (4) 4-knot natural splines. We then used Akaike information criterion (AIC) to select the final modeling approach. Similarly, AIC guided the multivariable modeling strategy with either natural 3-knot cubic splines or deciles to capture weekly temperature and absolute humidity. Lags of ±3 weeks between the exposure and outcome were investigated to accommodate potential delays between ARI and subsequent complications, including vascular events (eg, lags of 0 to +3 weeks [3, 5]), and because laboratory surveillance is assumed to reflect respiratory illness presenting in the community at an earlier time point (eg, lag = –2 weeks).

Results are presented as an incidence rate ratio (IRR) and corresponding 95% confidence interval (CI). The partial autocorrelation function for each model was investigated for evidence of residual autocorrelation, and where indicated, an additional lag term was included in the final model. The proportion of MI or stroke events attributed to each virus was estimated by predicting the number of events under the final model (*X*) and under a model assuming zero circulating virus (*Y*) as (*X − Y*) / *X*. We reestimated this value for weeks where levels of circulating virus were high (≥90th percentile of weekly viral counts).

### Sensitivity Analyses

We also examined the impact of excluding data for weeks prior to 2010 (approximately half the study period) because of concerns that health-seeking behavior and surveillance practices changed during and after the start of the 2009 influenza pandemic. Finally, we examined whether restricting viral respiratory infections to people aged ≥45 years substantially altered the interpretation of our results.

## RESULTS

Weekly counts of hospital admissions for MI averaged 476 per week (interquartile range [IQR], 429–553) for adults aged 45–64 years, 361 (IQR, 318–416) for those aged 65–74 years, and 513 (IQR, 443–583) in those aged ≥75 years (Table 1). The equivalent figures for stroke were 302 (IQR, 264–352), 303 (IQR, 264–354), and 564 (IQR, 496–682), respectively (Table 1).

**Table 1.** Description of Myocardial Infarction and Stroke Admissions, Viral Infections, and Environmental Data During the Period April 2004 to March 2015

Parameter	Total, No.	Weekly Median (IQR)
MI admissions		
MI admissions, age 45–64 y	280 890	476 (429–553)
MI admissions, age 65–74 y	209 346	361 (318–416)
MI admissions, age ≥75 y	296 261	513 (443–583)
Total MI admissions, age ≥45 y	786 497	1347 (1217–1541)
Stroke admissions		
Stroke admissions, age 45–64 y	177 505	302 (264–352)
Stroke admissions, age 65–74 y	178 454	303 (264–354)
Stroke admissions, age ≥75 y	337 035	564 (496–682)
Total stroke admissions, age ≥45 y	692 994	1175 (1023–1395)
Viral infections in all ages		
Adenovirus	16 282	23 (11–40)
Influenza	43 642	11 (5–53)
HMPV^a^	5850	13 (4–29)
Parainfluenza	17 672	22 (11–53)
Rhinovirus	43 408	55 (7–127)
RSV	85 639	27 (9–171)
Environmental data		
Maximum temperature, °C	…	14.2 (9.6–18.6)
Mean temperature, °C	…	10.3 (6.5–14.4)
Minimum temperature, °C	…	6.6 (3.1–10.5)
Absolute humidity, g/m^3^	…	7.6 (6.3–9.6)

Abbreviations: HMPV, human metapneumovirus; IQR, interquartile range; MI, myocardial infarction; RSV, respiratory syncytial virus.

^a^Data for January 2010 to March 2010.

**Figure 1.**
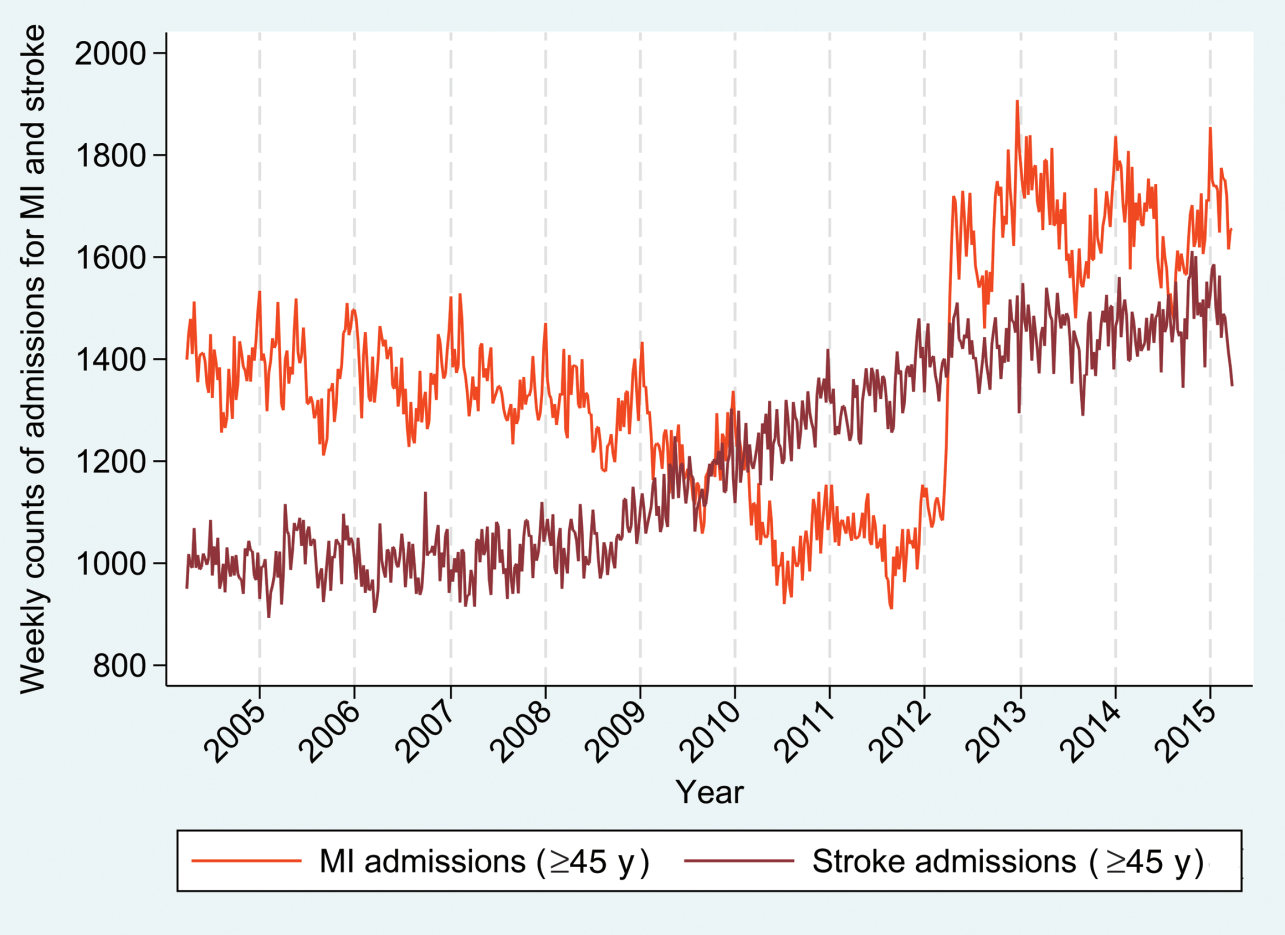
Weekly counts of myocardial infarction (MI) and stroke admissions in England for people aged ≥45 years.

There were strong temporal shifts in the frequency of MI and stroke, particularly for MI in 2012, which coincides with the introduction of the third universal definition of MI, reflecting the development of more sensitive assays for myocardial necrosis [17]. There was evidence of a winter peak for both MI and stroke, particularly within the ≥75 age group (Figure 1).

**Figure 2.**
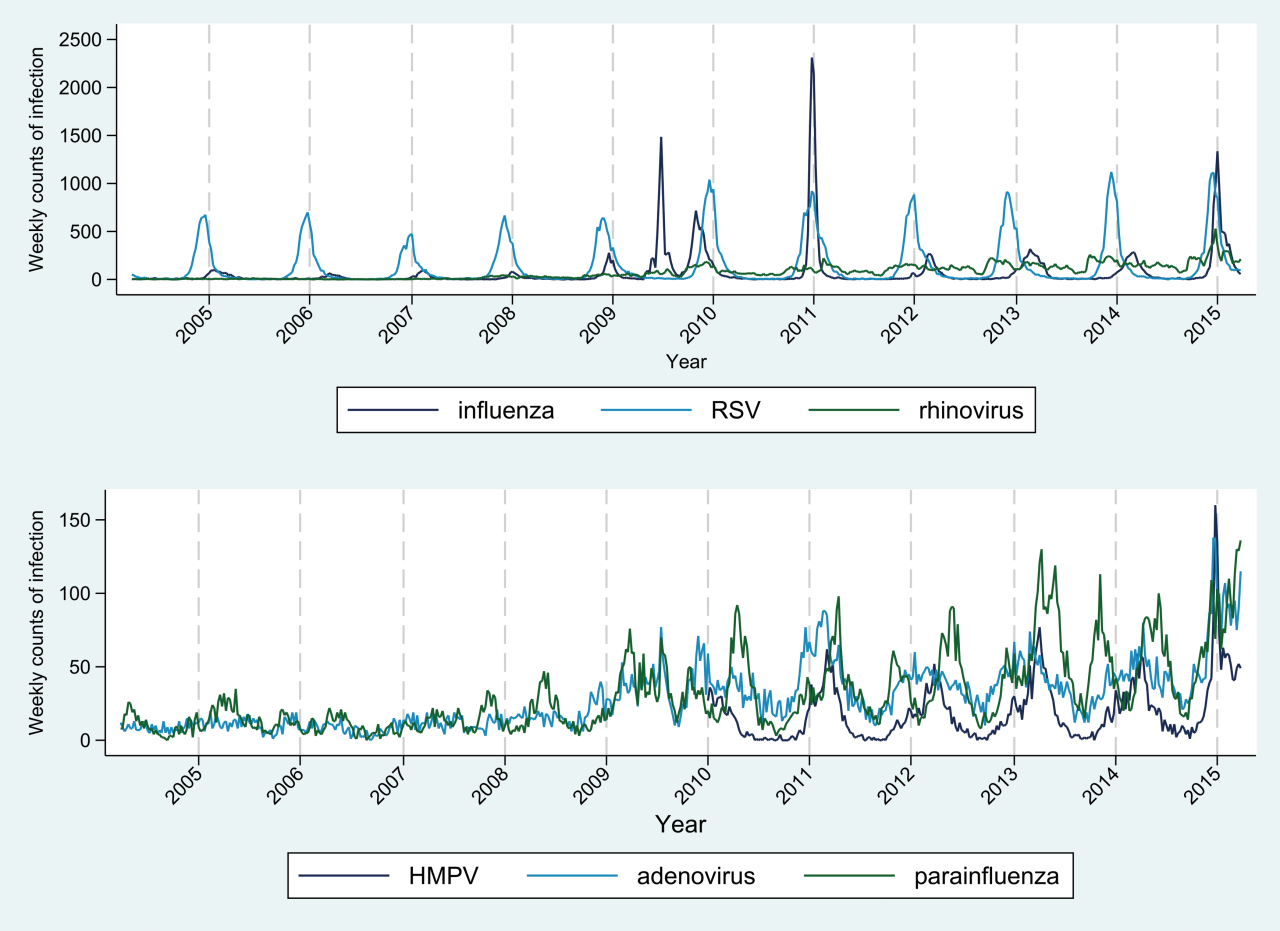
Weekly counts of laboratory-confirmed respiratory viruses between 1 April 2004 and 31 March 2015. Abbreviations: HMPV, human metapneumovirus; RSV, respiratory syncytial virus.

Weekly counts of all respiratory viruses in all ages are outlined in Figure 2 and Table 1, and range from a median of 11 cases per week (IQR, 5–53) for influenza to 55 (IQR, 7–127) for rhinovirus. Strong seasonal patterns with large fluctuations in weekly counts were evident for different viruses, particularly for RSV (IQR, 9–171).

Environmental data suggested minimum, mean, and maximum weekly temperatures of 6.6°C (IQR, 3.1°C –10.5°C), 10.3°C (IQR, 6.5°C –14.4°C), and 14.2°C (IQR, 9.6°C –18.6°C). Average weekly absolute humidity was 7.6 g/m^3^ (IQR, 6.3–9.6 g/m^3^).

### Association Between MI Admissions and Viral Respiratory Infection

The final multivariable models incorporated calendar quarter of MI admission, and deciles of maximum temperature and mean absolute humidity. In the adjusted models, there was no evidence of an association between weekly counts of MI for people aged 45–64 years and any of the respiratory viruses (*P* > .1 for all models). For MI in people aged 65–74 years, there were small but statistically significant positive associations with weekly counts of adenovirus (IRR, 1.00091 [95% CI, 1.00029–1.00153]), rhinovirus (IRR, 1.00029 [95% CI, 1.00009–1.00048]), and RSV (IRR, 1.00009 [95% CI, 1.00006–1.00013]): the best-fitting models were lagged by 3 weeks (Table 2). There was no evidence of an association between weekly infections with influenza, parainfluenza, or HMPV and MI admissions aged 65–74 years. In the ≥75 age group, associations remained similar to the age group 65–74 years for adenovirus, rhinovirus, and RSV (Table 2). However, additional associations were evident for influenza (IRR, 1.00006 [95% CI, 1.00003–1.00009]) and HMPV (IRR, 1.00146 [95% CI, 1.00096–1.00196]). Respiratory infection remained significant for influenza or HMPV in all models with lags of ±2 weeks, with the best-fitting models having 0–1 week lags.

**Table 2.** Associations Between Myocardial Infarction or Stroke Admission in the Final Model for Each Age Group and Virus

Outcome	Age Group, y	Virus	IRR	(95% CI)	*P* Value	Lag Period
MI admissions	≥75	Influenza	1.000056	(1.000027–1.000085)	<.0001	+1 week lag
		RSV	1.000102	(1.000072–1.000132)	<.0001	–3 week lag
		Adenovirus	1.000948	(1.000371–1.001525)	.001	–3 week lag
		Rhinovirus	1.000354	(1.000168–1.000540)	<.0001	–2 week lag
		HMPV	1.001460	(1.000964–1.001956)	<.0001	None
	65–74	Adenovirus	1.000911	(1.000288–1.001534)	.004	–3 week lag
		Rhinovirus	1.000285	(1.000090–1.000479)	.004	–3 week lag
		RSV	1.000092	(1.000059–1.000125)	<.0001	–3 week lag
All stroke admissions	≥75	HMPV	1.000863	(1.000420–1.001305)	<.0001	–1 week lag
		RSV	1.000046	(1.000021–1.000071)	.002	–3 week lag
Ischemic stroke admissions	≥75	Influenza	1.000051	(1.000027–1.000075)	<.0001	–1 week lag
		RSV	1.000064	(1.000036–1.000092)	<.0001	–3 week lag
		Adenovirus	1.000749	(1.000237–1.001261)	.004	–3 week lag
		Rhinovirus	1.000345	(1.000181–1.000509)	<.0001	–2 week lag
		HMPV	1.000671	(1.000232–1.001110)	.003	None

Models where there was no evidence of an association between respiratory infection and admission are not shown.

Abbreviations: CI, confidence interval; HMPV, human metapneumovirus; IRR, incidence rate ratio; MI, myocardial infarction; RSV, respiratory syncytial virus.

### Association Between All Stroke Admissions and Viral Respiratory Infection

As with MI, the final multivariable models incorporated calendar quarter of stroke admission, and deciles of maximum temperature and mean absolute humidity. In the adjusted models, there was no evidence of an association between weekly counts of any respiratory virus and stroke in people aged 45–64 years or 65–74 years (*P* > .1 for all models). Weekly counts of RSV (IRR, 1.00005 [95% CI, 1.00002–1.00007]) and HMPV (IRR, 1.000863 [95% CI, 1.00042–1.00131]) were positively associated with counts of stroke admissions aged ≥75 years in models with lags of ±2 weeks, with the best-fitting models having 1- to 3-week lags (Table 2). There was no evidence of an association between weekly counts of stroke aged ≥75 years and viral respiratory infection in the final models (those with the lowest AIC) for adenovirus, influenza, parainfluenza, or rhinovirus. In models with higher AICs (data not shown), there was evidence of a positive association between stroke admissions among persons aged ≥75 years and both influenza and rhinovirus infections, with lags of 1–2 weeks.

### Stratifying Stroke Admissions Into Ischemic and Hemorrhagic Types

Ischemic stroke accounted for 61%, 75%, and 81% of all stroke admissions in people aged 45–64, 65–74, and ≥75 years, respectively. Results for ischemic stroke in the ≥75 year age group closely mirrored those for MI, with small but significant positive associations identified for adenovirus (IRR, 1.000749 [95% CI, 1.000237–1.001261]), rhinovirus (IRR, 1.000345 [95% CI, 1.000181–1.000509]), RSV (IRR, 1.000064 [95% CI, 1.000036–1.000092]), influenza (IRR, 1.000051 [95% CI, 1.000027–1.000075]), and HMPV (IRR, 1.000671 [95% CI, 1.000232–1.00111]) (Table 2). There was no evidence of an association between respiratory virus infection and ischemic stroke in people aged 45–74 years, or with hemorrhagic stroke in any age group.

### Proportion of Events Attributable to Viral Respiratory Infection

**Figure 3.**
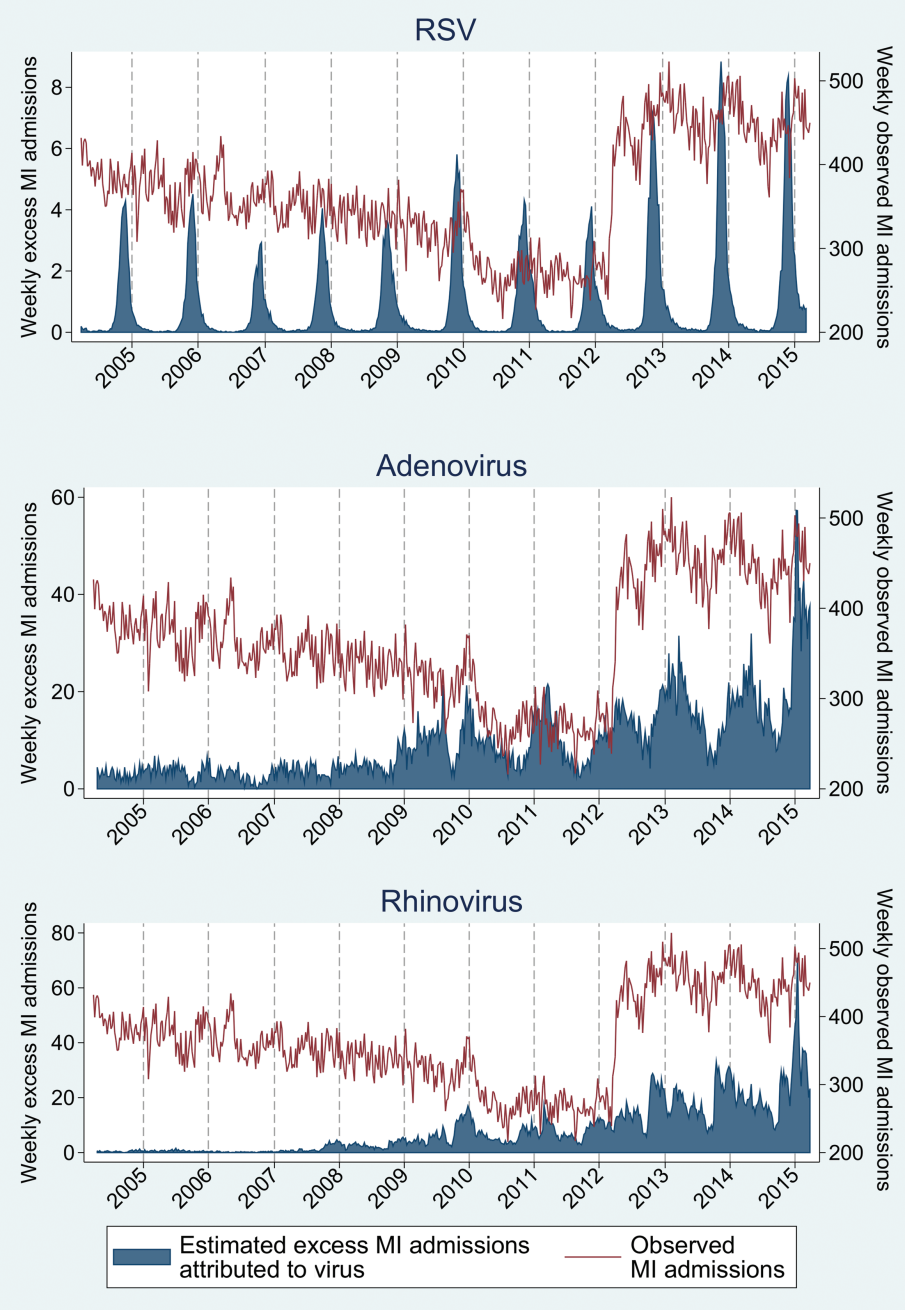
Weekly observed admissions for myocardial infarction in 65- to 74-year-olds, and the estimated excess admissions attributable to each virus. Abbreviations: MI, myocardial infarction; RSV, respiratory syncytial virus.

**Figure 4.**
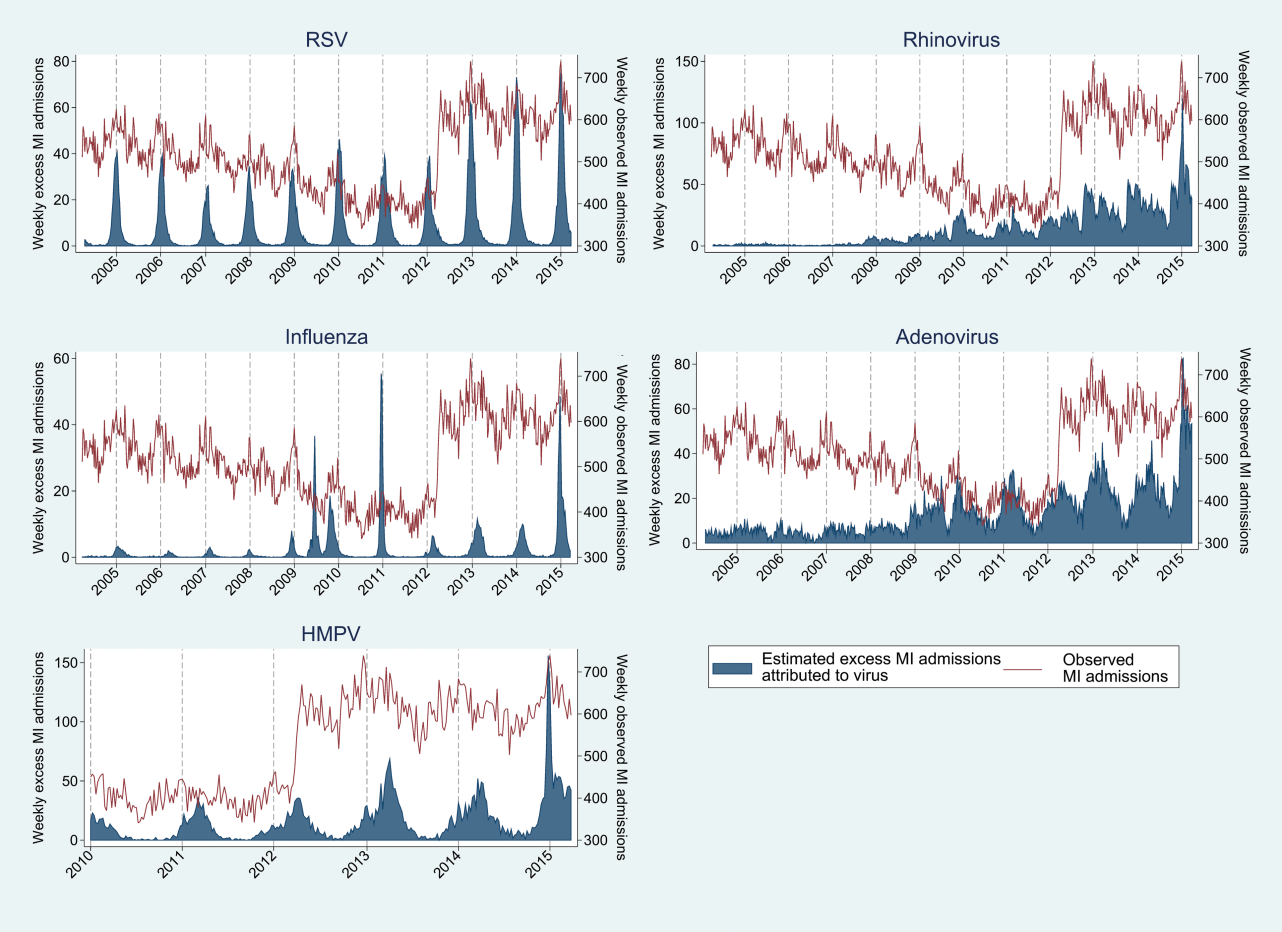
Weekly observed admissions for myocardial infarction in patients aged >75 years, and the estimated excess admissions attributable to each virus. Abbreviations: HMPV, human metapneumovirus; MI, myocardial infarction; RSV, respiratory syncytial virus.

**Figure 5.**
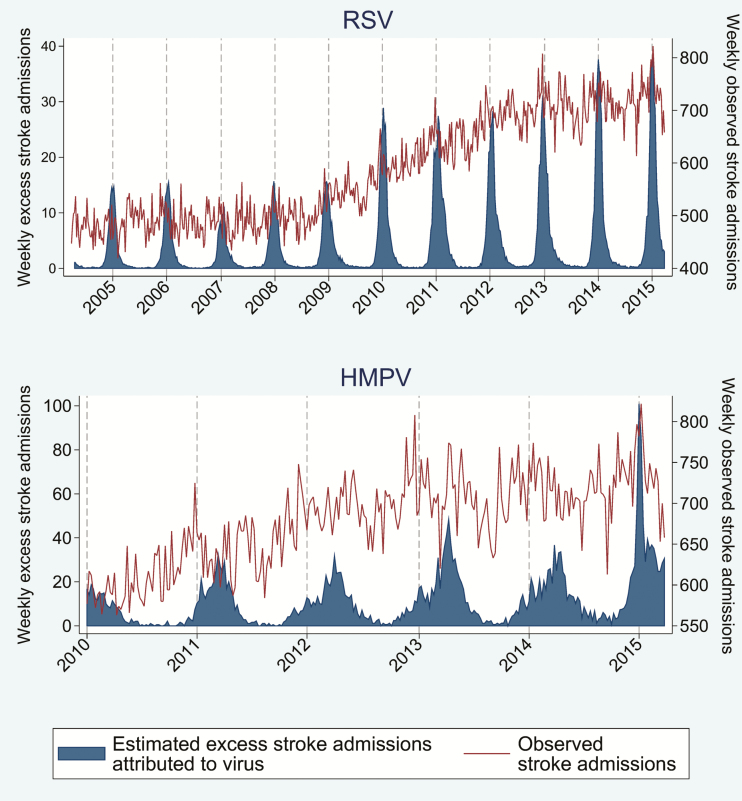
Weekly observed admissions for stroke in patients aged >75 years, and the estimated excess admissions attributable to each virus. Abbreviations: HMPV, human metapneumovirus; RSV, respiratory syncytial virus.

**Figure 6.**
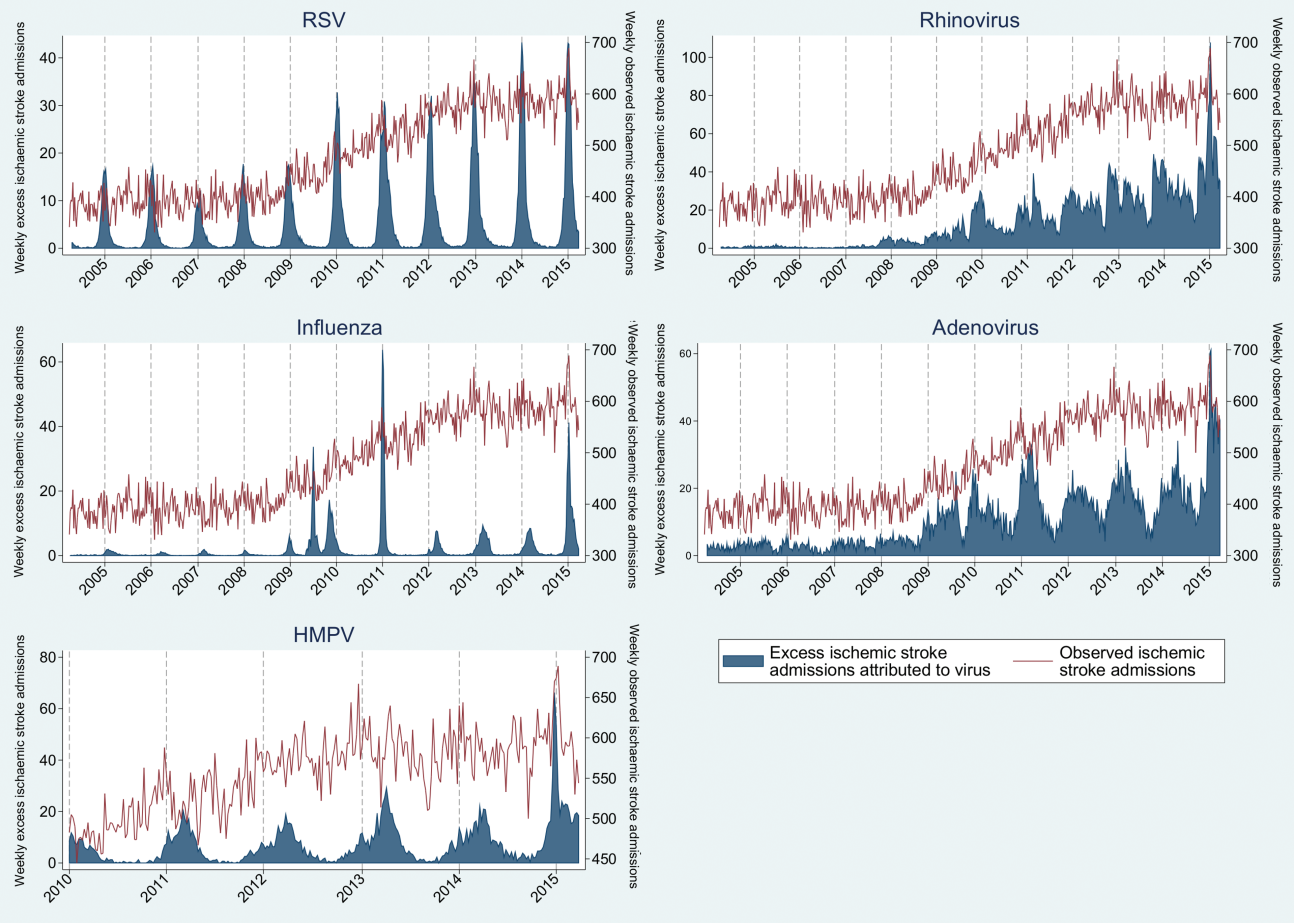
Weekly observed admissions for ischemic stroke in patients aged >75 years, and the estimated excess admissions attributable to each virus. Abbreviations: HMPV, human metapneumovirus; RSV, respiratory syncytial virus.

The proportion of events attributable to viral respiratory infection is outlined in Table 3. Figures 3–6 outline the observed and estimated weekly number of admissions attributable to viral respiratory infection for MI in persons aged 65–74 (Figure 3) and ≥75 years (Figure 4), and for all (Figure 5) and ischemic (Figure 6) stroke admissions aged ≥75 years. Across the whole study period, the proportion of MI admissions in people aged 65–74 years attributable to each virus was 1.4% for RSV, 2.0% for rhinovirus, and 2.6% for adenovirus. When restricted to weeks with a high burden of respiratory infection, the proportion of MI admissions attributable to infection increased to 6.3%–6.9%. Infection-attributable MI admissions were similar in those aged >75, namely 1.6% for RSV and 2.6% each for adenovirus and rhinovirus across the whole study period. Among high virus burden weeks, the proportion of MI admissions attributable to infection was 6.6%–7.6%. In addition, the proportion of MI admissions among those aged ≥75 years attributable to each respiratory virus was 3.0% for HMPV and 0.4% for influenza, and increased to 2.8%–9.2% when restricted to weeks with high virus burden. The proportion of all stroke admissions in persons aged ≥75 years attributable to infection was 0.7% for RSV and 1.7% for HMPV: These estimates increased to 4.1%–5.4% for weeks with high virus burden. The proportion of ischemic stroke admissions attributable to each respiratory virus was 0.4% (influenza), 1.0% (RSV), 2.3% (adenovirus), and 2.9% (rhinovirus) and increased to 2.6%–7.7% when restricted to weeks with high virus burden (Table 3).

**Table 3.** Proportion of Admissions for Vascular Events Attributed to a Given Viral Infection for All Weeks in the Study Period, and Restricted to Weeks With High (>90th Percentile) Counts of Infection

Outcome	Age Group, y^a^	Virus	Proportion of Vascular Events Attributed to Infection			
			2004–2015		2010–2015	
			All Weeks	Weeks With High Counts of Infection	All Weeks	Weeks With High Counts of Infection
MI admissions	≥75	Influenza	0.4%	2.8%	0.7%	4.3%
		RSV	1.6%	7.6%	2.0%	9.2%
		Adenovirus	2.6%	6.6%	5.7%	11.6%
		Rhinovirus	2.6%	7.6%	5.0%	9.6%
		HMPV^b^	…	…	3.0%	9.2%
	65–74	Adenovirus	2.6%	6.5%	5.3%	10.7%
		Rhinovirus	2.0%	6.3%	3.1%	5.8%
		RSV	1.4%	6.9%	1.7%	7.8%
All stroke admissions	≥75	RSV	0.7%	3.5%	0.9%	4.1%
		HMPV^b^	…	…	1.7%	5.4%
Ischemic stroke admissions	≥75	Influenza	0.4%	2.6%	0.6%	3.9%
		RSV	1.0%	4.9%	1.2%	5.7%
		Adenovirus	2.3%	5.3%	3.6%	7.3%
		Rhinovirus	2.9%	7.7%	4.6%	9.1%
		HMPV^b^	…	…	1.3%	4.2%

Abbreviations: HMPV, human metapneumovirus; MI, myocardial infarction; RSV, respiratory syncytial virus.

^a^Based on optimal model for each virus.

^b^Data for January 2010 to March 2015.

### Sensitivity Analyses

All associations between virus-specific counts of infection and MI or stroke admissions identified previously remained statistically significant after restricting data to time points after 2010. However, the proportion of admissions attributable to infection was higher relative to the whole time period (Table 3).

Restricting counts of weekly reports of viral respiratory infection to people aged ≥45 years did not meaningfully alter the associations reported previously for MI or stroke in people aged 45–64 and ≥75 years. However, for people aged 65–74 years, the association with MI remained for RSV, but was no longer significantly associated with adenovirus and rhinovirus infections.

## DISCUSSION

We examined temporal associations between weekly numbers of MI or stroke admissions and laboratory-confirmed viral respiratory infections. Our results show that, after controlling for environmental factors and long-term trends in admissions, higher circulating levels of some respiratory viruses coincide with increased MI and stroke admissions in older people. Specifically, MI and ischemic stroke admissions among those aged ≥75 years were associated with adenovirus, rhinovirus, RSV, influenza, and HMPV. MI admissions in the age group 65–74 years were associated with adenovirus, rhinovirus, and RSV, and hemorrhagic stroke admissions were not associated with respiratory infections for any age group. For the full study period, we estimate that a small but significant proportion of MI (0.4%–3.0%) and ischemic stroke (0.4%–2.9%) admissions in the elderly may be attributable to viral infection, with increased burden during weeks with high levels of reported respiratory infections (up to 7.7%). Conversely, we did not identify evidence of an association between circulating levels of any respiratory viruses and MI or stroke admissions in people aged <65 years.

Our results add to a growing body of evidence supporting a link between respiratory infection and acute ischemic events [5, 10, 18–25]. In accordance with other studies, we found that a small but significant proportion of ischemic event admissions among older adults may be attributable to influenza infection [5, 22, 26]. To the best of our knowledge, this is the first study examining the association between acute vascular events and a broad array of common respiratory viruses. Our results provide new evidence that the temporal association between ischemic vascular events and respiratory infection is not unique to influenza virus, but more widely to rhinovirus, adenovirus, RSV, and HMPV, which have similar clinical presentations [27]. By contrast, our results did not indicate an association between parainfluenza and ischemic vascular admissions in any age group. This finding may reflect a true lack of association or limited statistical power, or differences in seasonality between MI and parainfluenza type 3 (the major circulating type for England), which has a spring/summer peak [28]. Our results for stroke admissions differ to a Canadian time-series study that identified positive associations between weekly ARI consultations with both hemorrhagic (1- to 3-week lag period) and ischemic stroke (16-week lag period) admissions [29]. However, the age groups, lag periods investigated, and local seasonality of infection are not directly comparable between the studies.

Several mechanisms are thought to link infection and acute vascular events, including release of proinflammatory cytokines, disruption of atherosclerotic plaques, and physiological impacts on heart rate and vasoconstriction [30]. However, much of this evidence examines thrombotic events, with less consistent evidence for hemorrhagic events [29, 31–35]. A higher prevalence of preexisting cardiovascular and chronic conditions such as cancers, inflammatory diseases, and respiratory diseases in the elderly is likely to underpin the greater risk of infection-triggered events in this group [36–38]. Due to the ecological nature of our study, we were unable to examine the impact of such comorbidities and other confounders (such as influenza vaccination [24] or treatment with antivirals [39]) that are likely to mediate the risk of vascular events through infection.

Our study is the first in England to investigate acute vascular events attributable to a broad array of viruses (adenovirus, HMPV, parainfluenza, rhinovirus, influenza, and RSV). The main strength of this study is the use of laboratory-confirmed infections with national coverage, over an 11-year period, and addressing confounding by environmental factors. The key limitation is the use of ecological methods, which cannot infer causality. However, these methods were well suited to the research question and data sources: The specificity of respiratory infections reported via this national surveillance scheme is likely to exceed 90% [36]. In contrast, counts of ARI will be underestimated due to lower sensitivity of testing (eg, 47%–80%) [36] and highly incomplete sampling, particularly of community settings. Likewise, we chose to focus on MI and stroke because these have clearer diagnostic criteria than other types of vascular event (eg, angina) and are thus less prone to misclassification.

Time-series methods do not rely on having a high proportion of cases diagnosed, provided that the proportion recorded does not vary radically over time. We anticipated that ascertainment of respiratory infections would be higher during the summer wave of the 2009 influenza pandemic (tending to bias our estimate of the true association with hospital admissions toward the null; particularly for influenza [Figure 2]). However, overall, our study results showed similar patterns of association (but with differences in the magnitude of association) across the whole time period and for time points after 2010, suggesting that changes in testing during and after the pandemic do not substantially impact on the interpretation of our findings. We used AIC as an objective aid for selecting the final analysis models; however, the selection of one process over another remains contested and might influence the results [37].

Our results implicate a wider range of respiratory viruses (RSV, HMPV, influenza, adenovirus, and rhinovirus) as potential triggers of ischemic vascular events in older people, which has important implications for clinical management. Of these viruses, currently only influenza has an effective vaccine available, yet its effectiveness is unfortunately diminished in older age groups. RSV vaccines and antivirals are in development and currently being evaluated in clinical trials. Further evidence is needed on the potential harms and benefits of strategies drawing on antiviral, anti-inflammatory, or antithrombotic agents, such as low-dose aspirin for reducing the risk of vascular events following infection. Such approaches may also lead to better understanding of the underpinning mechanisms. In the United Kingdom, adults aged <65 years are not eligible for free influenza vaccination unless they are in a clinical risk group. The absence of an association between respiratory infection and vascular events in people aged 45–64 years therefore lends support to current age- and risk factor–focused influenza vaccination strategies. Future work should explore pathogen-specific vascular triggers, including bacterial respiratory infection [38, 40–42].

## CONCLUSIONS

We showed small but strongly significant associations between respiratory infection and MI and ischemic stroke hospitalizations in the elderly. These associations occurred across a range of respiratory viruses (RSV, HMPV, influenza, adenovirus, and rhinovirus), thus highlighting the importance of further evaluation of the impact of antivirals, vaccination, and antithrombotic agents around the time of infection on cardiovascular outcomes.

## Notes

***Acknowledgments.*** We are very grateful for all hospital laboratories in England who submitted their data to the LabBase data system, making it possible for us to conduct this study.

***Financial support.*** This work was supported by an Academy of Medical Sciences Starter Grant for Clinical Lecturers (to C. W. G.). C. W. G. is supported by a Wellcome Intermediate Clinical Fellowship (grant number 201440_Z_16_Z). We also acknowledge the support from the Farr Institute of Health Informatics Research (Medical Research Council grant number London MR/K006584/1).

***Potential conflicts of interest.*** All authors: No reported conflicts of interest. All authors have submitted the ICMJE Form for Disclosure of Potential Conflicts of Interest. Conflicts that the editors consider relevant to the content of the manuscript have been disclosed.
